# Supplementary material for: Classifying acoustic signals into phoneme categories: average and dyslexic readers make use of complex dynamical patterns and multifractal scaling properties of the speech signal
Source: PeerJ. 2015 Mar 26;3:e837. doi: 10.7717/peerj.837 (PMC4380160; doi:10.7717/peerj.837)
Supplement: Table S1 [file peerj-03-837-s001.docx]

| Table S1 Residual norms and SDs of the MF-DFA simple linear regression fit. | | | | | | | | |
| --- | --- | --- | --- | --- | --- | --- | --- | --- |
|  | **Acoustic Manipulation** | | | | | | | |
|  | *None* | | *Slowed Down* | | *Amplified* | | *Both* | |
| **Stimulus** | res norm | SD | res. norm | SD | res norm | SD | res norm | SD |
| /bAk/ | 2.56 | 0.51 | 2.76 | 0.82 | 2.76 | 0.68 | 2.14 | 0.38 |
| 2 | 2.60 | 0.61 | 2.05 | 0.41 | 2.78 | 0.75 | 2.20 | 0.48 |
| 3 | 2.57 | 0.62 | 2.57 | 0.96 | 2.72 | 0.73 | 2.13 | 0.43 |
| 4 | 2.54 | 0.63 | 2.48 | 0.91 | 2.70 | 0.74 | 2.03 | 0.37 |
| 5 | 2.51 | 0.64 | 2.46 | 0.97 | 2.61 | 0.67 | 1.91 | 0.34 |
| 6 | 2.49 | 0.65 | 2.69 | 1.01 | 2.59 | 0.68 | 1.86 | 0.34 |
| 7 | 2.48 | 0.66 | 2.54 | 0.91 | 2.54 | 0.63 | 1.83 | 0.33 |
| 8 | 2.46 | 0.67 | 2.38 | 0.73 | 2.52 | 0.62 | 1.93 | 0.35 |
| 9 | 2.45 | 0.67 | 2.58 | 1.00 | 2.51 | 0.62 | 1.95 | 0.35 |
| /dAk/ | 2.44 | 0.68 | 2.27 | 0.67 | 2.49 | 0.61 | 1.92 | 0.34 |
