# Supplementary material for: Classifying acoustic signals into phoneme categories: average and dyslexic readers make use of complex dynamical patterns and multifractal scaling properties of the speech signal
Source: PeerJ. 2015 Mar 26;3:e837. doi: 10.7717/peerj.837 (PMC4380160; doi:10.7717/peerj.837)
Supplement: Supplemental Information 1 [file peerj-03-837-s002.doc]

- [Consent form_3   competent 12-17](http://www.ru.nl/publish/pages/532759/ciemo_3_toestemmingsverklaring.doc)

**MODEL 3**

**CONSENT FORM***

for participation in the research project:

... *(title of the research project)*

**FOR THE MINOR:**

• The research was explained to me. I read the letter about the research (*version code:* ...) carefully.

I asked the questions I had about the research. I have thought hard about taking part in the research, and it is my own free choice to take part. I may stop taking part in the research whenever I want.

**• I would like to take part in the research.**

Name:

Date:

Signature: Date:

**FOR THE PARENTS/GUARDIAN:**

• I hereby confirm that I was satisfactorily informed about the research and that I have read and understood the information sheet (version code: ... ). I was allowed sufficient time to consider whether to give my consent and was also given the opportunity to ask questions. Any questions I asked were answered to my satisfaction. I know I may withdraw my consent at any time without giving a reason.

**• I hereby give my consent for the above-mentioned person to be included as participant in the research.**

Surname and initials: Surname and initials:

Relation to the participant : Relation to the participant:

Signature: Signature:

Date Date:

-------------------------------------------------------------------------------------------------------------------------------------

• The undersigned declares that the persons named above have been informed both in writing and in person about the aforementioned research. He/she also declares that the person named above may prematurely terminate their participation with no consequences for this person.

Name:

Position:

Signature: Date:

-------------------------------------------------------------------------------------------------------------------------------------

** This form is for research involving competent human subjects aged 12-17. This type of research requires consent from both the minor and the parents who have custody of the minor or the minor’s guardian.*
