# Supplementary material for: Classifying acoustic signals into phoneme categories: average and dyslexic readers make use of complex dynamical patterns and multifractal scaling properties of the speech signal
Source: PeerJ. 2015 Mar 26;3:e837. doi: 10.7717/peerj.837 (PMC4380160; doi:10.7717/peerj.837)
Supplement: Supplemental Information 2 [file peerj-03-837-s003.doc]

- [Consent form_4    under 12 (incl. chance finding clause)](http://www.ru.nl/publish/pages/532759/ciemo_4_toestemmingsverklaring_toeval.doc)

**MODEL 4**

**CONSENT FORM***

for participation in the research project:

... *(title of the research project)*

**FOR THE PARENTS/GUARDIAN:**

My consent was requested for the following person to be included as participant in the above-mentioned research project:

Surname and initials:

Date of birth:

• I hereby confirm that I was satisfactorily informed about the research,** and that I have read and understood the information sheet (version code: ... ). I was allowed sufficient time to consider whether to give my consent and was also given the opportunity to ask questions. Any questions I asked were answered to my satisfaction. I know I may withdraw my consent at any time without giving a reason.

**• I hereby give my consent for the above-mentioned person to be included as participant in the research.**

Surname and initials: Surname and initials:

Relation to the participant: Relation to the participant:

Signature: Signature:

Date: Date:

-------------------------------------------------------------------------------------------------------------------------------------

• The undersigned declares that the persons named above have been informed both in writing and in person about the aforementioned research. He/she also declares that the person named above may prematurely terminate their participation with no consequences for this person.

Name:

Position:

Signature: Date:

------------------------------------------------------------------------------------------------------------------------------------

** This form is for research involving minors under the age of 12.*

*** In exceptional cases new information concerning your health may be obtained. In such cases you will be informed about this by the researcher. If you do not wish to be informed about this, you cannot take part in the research. You should also know that the obtained research data will not be considered from a medical perspective. Your participation in this research can therefore not be deemed to be a medical test.*
